# Global soybean trade intensifies the impacts of dietary transition on human mobility

**DOI:** 10.1016/j.isci.2025.112426

**Published:** 2025-04-15

**Authors:** Nan Jia, Hongbo Yang, Xin Lan, Yinshuai Li, Yongze Song, Zehua Zhang, Wen Song, Rui Zhao, Tianwu Ma, Ruishan Chen

**Affiliations:** 1 School of Design, Shanghai Jiao Tong University, Shanghai 200240, China; 2 State Key Laboratory of Urban and Regional Ecology, Research Center for Eco-Environmental Sciences, China Academy of Sciences, Beijing 100085, China; 3 Department of Geography, Environment, and Spatial Sciences, Michigan State University, East Lansing, MI 48824, USA; 4 Environmental Science and Policy Program, Michigan State University, East Lansing, MI 48824, USA; 5 School of Design and the Built Environment, Curtin University, Bentley, Western Australia 6102, Australia; 6 College of Environmental Science and Engineering, Tongji University, Shanghai 20092, China; 7 Key Laboratory of Virtual Geographic Environment (Nanjing Normal University), Ministry of Education, Nanjing 210023, China; 8 Jiangsu Center for Collaborative Innovation in Geographical Information Resource Development and Application, Nanjing 210023, China; 9 School of Geography, Nanjing Normal University, Nanjing 210023, China

**Keywords:** Agricultural science, Sustainability aspects of food production

## Abstract

Soybeans have experienced massive growth in trade due to diet transitions. Large imports reduce expected returns for soybean growers in importer countries, influencing occupational choices and potentially facilitating population mobility, though the distant correlation’s impact remains unclear. We develop a framework integrating diet dynamics, trade indexes, and human mobility. We found: (1) dietary transition promotes human mobility through global soybean trade; (2) rural areas contribute far less than urban areas, illustrating decoupling between rural regions and international trade; (3) enhancing rural-trade coupling could improve dietary and crop flow in rural regions; (4) the study provides a new perspective on how dietary transition promotes human mobility. These findings help policymakers identify soybean trade strategies for socioeconomic development and formulate interventions to optimize population distribution. By addressing rural decoupling, we emphasize aligning rural areas with global trade to mitigate mobility pressures and socioeconomic disparities, without altering original terms.

## Highlights

• Dietary transitions drive human mobility through global soybean trade dynamics
• Urban diets contribute more to soybean trade-induced migration than rural diets
• Decoupling of rural areas from trade limits local agricultural-economic integration
• Integrating rural trade can optimize dietary systems and mitigate migration pressures

## Graphical abstract

**Figure:**
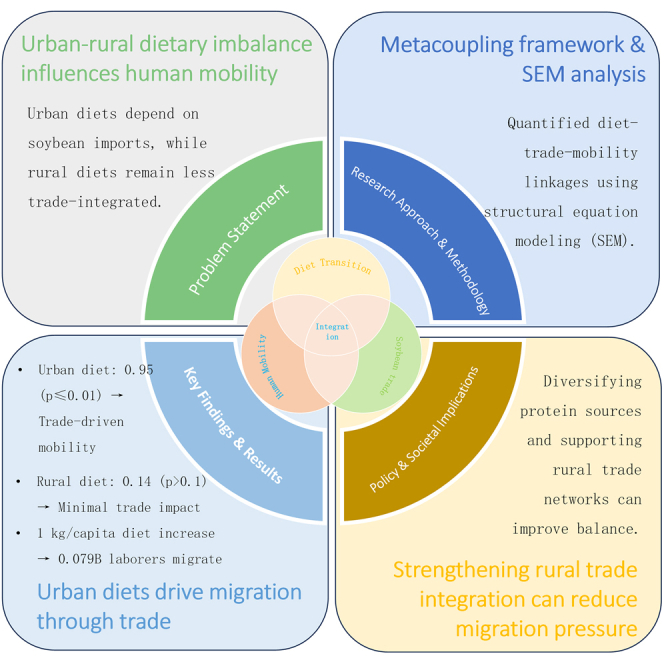

## Introduction

Human dietary transitions are a critical component of socioeconomic and environmental change. As global populations shift toward more protein-rich diets, the demand for agricultural products has escalated, reshaping trade flows, and driving human mobility. The global soybean trade, a key driver of these transitions, is a prime example of how dietary shifts can influence economic and social dynamics. The global soybean trade, a key driver of these transitions, is a prime example of how dietary shifts can influence economic and social dynamics. Soybeans, essential for protein, feed, and oil, have become a cornerstone of global agricultural trade, profoundly impacting both importing and exporting countries.^1^^,^^2^^,^^3^ Among importers, the influx of competitive imported products can reduce income for local growers, exacerbate income disparities, and drive migration patterns, particularly in rural regions.^4^^,^^5^ Exporters, on the other hand, experience expanded farmland use, attracting seasonal workers and reshaping local economies.^6^

China, as the world’s largest soybean importer, accounts for over 60% of global imports, creating profound social and environmental linkages with exporting countries, such as the United States, Brazil, and Argentina.^2^ This dominance in the soybean trade makes China an exemplary case for studying the broader impacts of dietary transitions. Its rapid urbanization and industrialization have widened income disparities between agricultural and non-agricultural sectors, leading to significant rural-to-urban migration and changes in household income structures.^7^ Despite these trends, the disproportionate impacts of dietary transitions and trade on rural populations remain underexplored. At present, over 250 million people migrate annually in China, driven by various socioeconomic factors. This underscores the need for a deeper understanding of the interplay between dietary transitions, trade, and human mobility. At present, over 250 million people migrate annually in China, driven by various socioeconomic factors. This underscores the need for a deeper understanding of the interplay between dietary transitions, trade, and human mobility.

While studies have examined the drivers of dietary transitions, trade liberalization, and migration independently, limited research addresses their interconnections, particularly within the context of specific crop trades like soybeans.^8^^,^^9^^,^^10^ Studies have analyzed the potential of global trade to reduce food inequality and generate unexpected benefits for impoverished regions.^11^^,^^12^ However, a deeper understanding of how dietary transitions influence the spatial distribution of soybean trade and human mobility is essential for management agencies to design better interventions. This gap hinders the development of targeted interventions to facilitate dietary transitions while addressing socioeconomic disparities through improved labor distribution.

The metacoupling framework, an extension of the telecoupling framework, provides a comprehensive approach to analyzing interconnected systems across spatial scales (Figure S1).^13^^,^^14^ This framework emphasizes five core components: systems (e.g., countries, regions), flows (e.g., commodities, information), agents (e.g., traders, policymakers), causes, and effects.^15^ Recent studies have demonstrated the utility of the telecoupling and metacoupling frameworks in addressing the integrate of socioeconomic and environmental issues on different spatial scales. For instance, researchers employed the telecoupling framework to reveal the cascading impacts of the Russia-Ukraine war on global trade, cropland expansion and biodiversity, illustrating how geopolitical conflicts can reshape distant food systems and ecological processes.^16^^,^^17^ Similarly, Kılkış et al. highlighted the interconnected effects of city-company collaborations on reducing pressures on Earth system boundaries, emphasizing the potential of coordinated actions to address global sustainability challenges.^18^ Furthermore, Li et al. investigated the effectiveness of global protected areas in resisting habitat loss using the metacoupling framework, shedding light on the spatial and temporal challenges of conservation strategies.^19^ Additionally, Liu et al. analyzed cross-provincial cultural ecosystem service flows in China’s metacoupled systems, highlighting how telecoupling and pericoupling dynamics vary across geographic regions, offering new insights into sustainable tourism and cultural ecosystem services.^20^ In this study, we apply the metacoupling framework to explore the connections between dietary transitions, global soybean trade, and human mobility. Global soybean exporters, such as Brazil and the United States, represent the sending systems, while China, as the world’s largest soybean importer, serves as the receiving system. Within the intracoupled system, human mobility in China’s primary soybean-producing province, Heilongjiang, exemplifies the localized impacts of trade (Figure S2). By integrating the metacoupling framework with structural equation modeling (SEM), this study quantitatively explores the pathways through which dietary transitions influence human mobility via global soybean trade. Specifically, the research investigates how urban dietary shifts contribute more significantly to this relationship than rural transitions, revealing a decoupling between rural regions and international trade. The framework not only underscores these disparities but also offers strategies to enhance rural integration into global trade, aiming to improve dietary and crop flow in these regions. Using SEM, we systematically assess the linkages between diet structure, soybean trade, and human mobility, focusing on China as the case study to address these interactions in a telecoupled global trade context. We aim to answer the following questions.

(1) How does dietary transition affect human mobility through soybean trade?
(2) Does dietary transition contribute to population mobility to the same extent in urban and rural areas?

## Results

### Trend analysis of data

**Figure 1:**
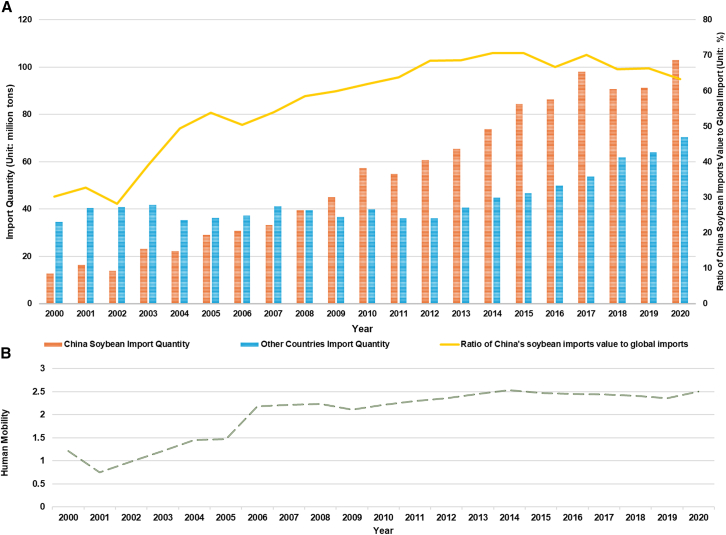
Changes in China’s soybean trade network between 2000 and 2020 (A) Soybean trade flow to China in 2000; (B) Soybean trade flow to China in 2020. Exporting countries increased from 8 to 23. Export quantities ranged from 182 tons to 2.82 million tons in 2000 and from 0.2 million tons to 6.06 million tons in 2020. Percentages indicate the share of total Chinese soybean imports from each country. All country names are shown in ISO.

Table 1 shows the information from the descriptions of the variables used in this study. In terms of diet composition, protein-related food consumption in urban and rural areas showed a continuously increasing trend from 2000 to 2020, while the consumption trend of other food items in urban areas was essentially flat, with only a small long-term increasing trend. The soybean import data show a gradual growth trend, from 26.94% of the world’s total soybean imports in 2000 to 50.02% in 2008, to 70.68% in 2015. Affected by the negative impacts of the Sino-US trade war, soybean trade showed a downward trend in 2018 and gradually increased in the following two years (Figure 1A).

**Table 1:** Descriptive information of variables for SEM

Class	Variables	Description
Diets	Rural protein containing	Consumption of protein-containing food in rural regions (unit: kg/per capita/yr)
	Urban protein containing	Consumption of protein-containing food in urban regions (unit: kg/per capita/yr)
	Others	Consumption of other food in urban regions (unit: kg/per capita/yr)
Trade	Import quantity	China’s total soybean imports from global exporters (unit: million tons/yr)
	Trade value	China’s total soybean imports value from global exporters (unit: million $/yr)
	Income gap	China’s urban-rural income gap (unit:$/per capita)
	The negative value of soybean production	Negative China’s soybean production (unit: million tons)
Human mobility	Total mobile population	Total mobile population in China (unit: 0.1 billion person/yr)

In general, the quantity of imported soybeans in China has shown a relatively stable trend after 2010 (60% of the world’s soybean exports). The total migrant population in China has shown a fluctuating trend of increase over 21 years, with brief decreases likely driven by temporary government policies (controls or subsidies for soybean cultivation) (Figure 1B).

### Exclusion of economic variables due to severe multicollinearity

The multicollinearity analysis results revealed that these economic variables exhibited extremely high multicollinearity, as indicated by their variance inflation factor (VIF) values: GDP (VIF = 2,185.01), GDPPRI (VIF = 1,171,242), and Agri_Pro (VIF = 1,260,515). Additionally, other variables, such as income gap_total (VIF = 38.53) and soybean production_local (VIF = 37.31) were also highly correlated with these economic indicators. The high VIF values suggest significant overlap and redundancy, particularly with the income gap variable already included in the model. This redundancy can inflate standard errors, distort coefficient estimates, and reduce model interpretability.

Based on these findings, we determined that including GDP, GDPPRI, and Agri_Pro as control variables would likely introduce instability and reduce the reliability of the regression results. As a result, these variables were excluded from the final model to ensure robustness and accuracy. This decision was based on rigorous statistical evaluation and aligns with best practices for minimizing multicollinearity in econometric models.

### Diet increase local human mobility through trade

**Figure 2:**
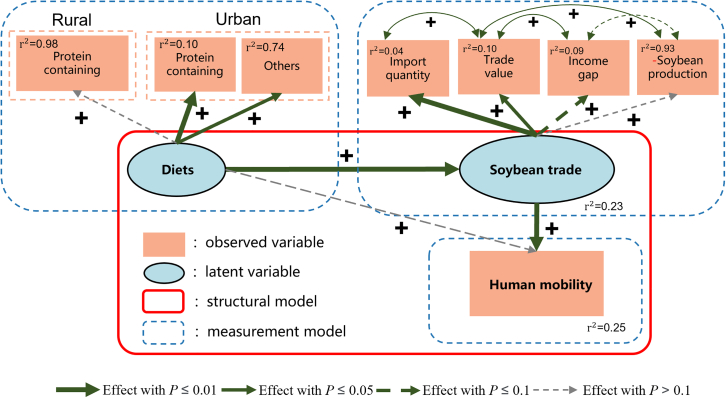
Conceptual model of the dietary transition mechanism affecting human mobility The transition to high-protein diets increases soybean import demand, decreases domestic soybean profitability, widens the rural-urban income gap, and drives human mobility.

The effect of each pathway through which the diets affected human mobility through trade is shown in Figure 2. Of the three latent variables comprising the diet, only rural protein-containing was not statistically significant (*p* > 0.1) in SEM. The protein food produced as a result of rural poultry farming and soybean cultivation could offset the soybean trade facilitated through purchasing behavior. Therefore, the effect of its path is 0.14. Food consumption within urban areas contributed more to diet, with urban protein-containing food correlating with diet at 0.95 and with urban other food at 0.51. This illustrates that when the consumption of protein-related foods and other foods grows by the same amount, protein-related food changes will make a greater contribution to trade volumes.

**Figure 3:**
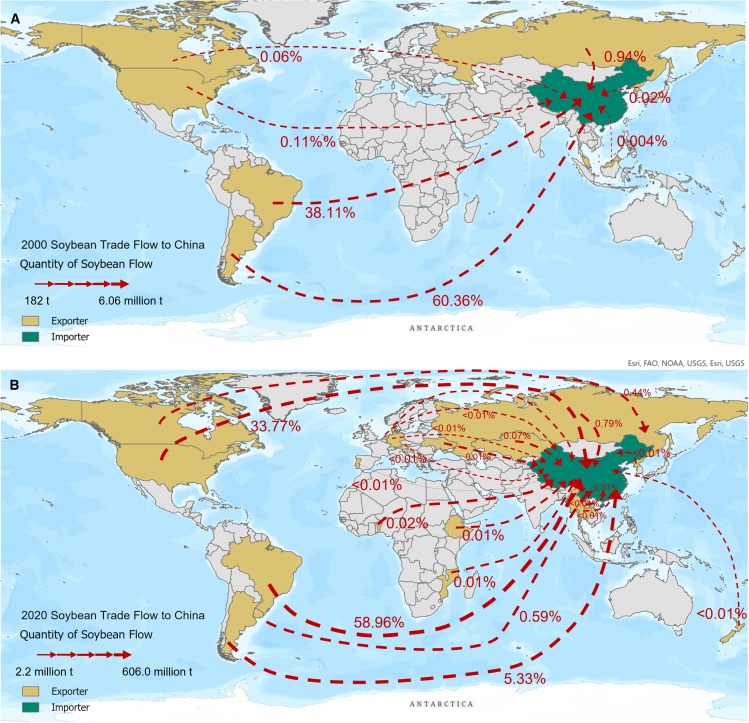
Illustration of the assumed linkages between diets, trade, and human mobility (A) Soybean trade flows to China in 2000. Red dashed arrows represent the trade routes, with arrow width proportional to the quantity of soybean flow. (B) Soybean trade flows to China in 2020. Numbers along the arrows indicate the proportion of total trade flow from each country to China, expressed as a percentage. The flow size scale ranges from 182 tons to 6.06 million tons in 2000 (A), and from 2.2 million tons to 606.0 million tons in 2020 (B). Countries are colored according to their role as exporters (yellow) or importers (green). Note: Solid arrows represent hypothesized causal pathways. “+” and “–” indicate the direction of effects.

**Figure 4:**
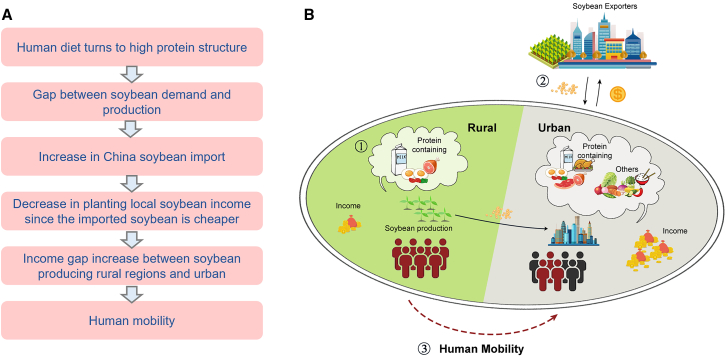
China’s soybean trade and human mobility trends from 2000 to 2020 (A) Soybean import quantities by China and other countries, with China’s import share of global trade. (B) Human mobility trends over the same period. Data points represent annual values; no error bars or statistical dispersion measures are shown.

**Figure 5:**
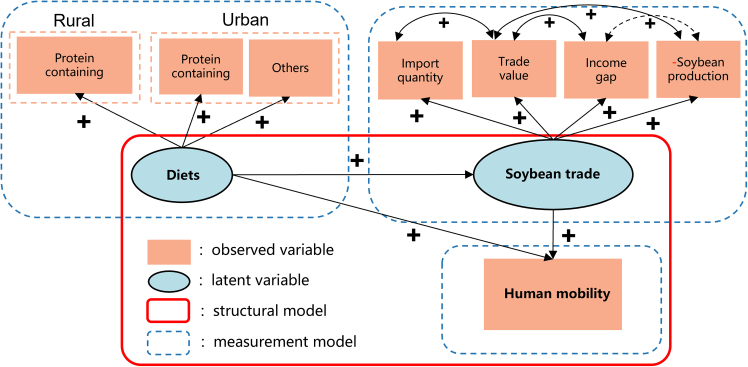
Visualization of the structural equation model results (Note: “+” and “−” represent assumptive positive and negative effects of the linkage, respectively.) Solid and dashed arrows indicate the strength and significance of the modeled relationships from the structural equation model (SEM). Line styles represent significance levels: solid dark green (*p* ≤ 0.01), solid light green (*p* ≤ 0.05), dashed green (*p* ≤ 0.1), and gray dashed (*p* > 0.1). Observed variables are shown in orange rectangles; latent variables in blue ellipses. Structural and measurement models are outlined in red and blue dashed lines, respectively.

The relationship and significance of each linkage in the assumed framework are shown in Figure 2 and Table S4. Diet has a significant positive effect on human mobility through trade. As with our assumption, diet transition did have a positive effect on human mobility through international soybean trade. For the three latent variables of diet, the structure of urban diet (protein-containing food and others) and rural protein-containing food both positively influenced diets, with urban’s food consumption *p* < 0.001, rural’s food *p* value was greater than 0.1. All four latent variables of trade positively related to the observed variable, except soybean production. The other three latent variables passed the significance test of *p* < 0.1. In addition, the effect of diet on human mobility captured by unspecified paths was positive but very weak (0.09). Although it was statistically insignificant, this unspecified pathway represented all other processes not covered in this study.

The linkage between diet, trade, and human mobility constitutes a different way in which diet affects human mobility. The growth of urban and rural dietary demand has contributed to urban-rural income differences, soybean import quantity, import value, and increased local soybean production. These latent variables of trade re-affected the dynamics in China’s human mobility.

The total effect of the diets on human mobility is 0.79%, which suggests that a 1% increase in the percentage of diet needed would increase human mobility by 0.79%. A 1 kg/per capita diet increase will prompt 0.079 billion laborers to migrate away from home to search for a higher income. If the impact was broken down, our results demonstrated that if urban and rural areas grow by the same amount of dietary consumption, the growth in urban areas will drive more people out of their homes from rural areas.

## Discussion

We analyzed the pathways through which dietary transitions, driven by global soybean trade, impact human mobility, with a particular focus on the differing contributions of urban and rural areas and the resulting policy implications for socioeconomic development. Our study and results contribute new insights to the “goal 2: zero hunger”, “goal 8: decent work and economic growth”, and “goal 12: responsible consumption and production” of the sustainable development goals (SDGs) of the United Nations reflect the international concern about food security, nutritionally balanced, and a decent income.^21^ To achieve these goals, scientists, policymakers, and practitioners need to understand how the diet transition affects local human mobility who are searching for a higher income through global soybean trade. Our study indicates that an integrated analysis of the paths among diet, trade, and human mobility can determine how the telecoupled regional diet affects local human mobility.

### Unraveling the impact of diet on human mobility

Changes in urban diets shape global trade patterns, which in turn affect human mobility between urban and rural. While the direct effect of diets on human mobility was not statistically significant, the indirect effect, through their influence on international soybean trade networks, was highly significant. The impact was driven by urban dietary changes, which fueled increased global soybean trade. Income disparity and soybean production emerged as key drivers, collectively contributing to increased human mobility (*p* < 0.01) (Figure 2). The dietary transition, particularly the increased demand for protein-containing foods, has driven continuous growth in the global soybean trade, reducing income from indigenous soybean cultivation and further prompting a significant shift in labor migration to non-agricultural sections to fill the income gap.

Our study underscores a critical yet usually overlooked mechanism: the growing demand for protein-rich diets lead to far-reaching socio-economic impacts. The Metacoupling framework helped us reveal previously unseen linkages between urban dietary transitions, global trade, and human mobility. Our findings extend existing studies, such as those exploring the impact of dietary shifts on virtual land imports,^22^ on carbon footprints,^23^ and on biodiversity.^24^ These impacts explored in previous studies represent more direct effects and are often the factors most commonly considered by policymakers. Our integrated perspective reveals the cascading effects of dietary transitions on socio-economic systems and emphasizes the need for targeted policies to mitigate the pressures associated with dietary transitions and labor migration. For instance, our findings indicate that declining income from indigenous soybean cultivation has driven significant labor migration to non-agricultural sectors. Therefore, implementing agricultural subsidies, as proposed by Fan et al.,^25^ may help stabilize rural incomes and alleviate the resulting migration pressures by addressing these economic vulnerabilities.

### Urban-rural dietary contributions and trade dynamics

In the present study, each latent variable contributed differently to the observed variables. In the composition of diets, we found variability in the degree of contribution of urban and rural areas to the diets. In a realistic process, yard farming and soybean cultivation reduce the trade contribution of changes in protein-containing foods in rural areas, which may explain our results showing that rural protein-containing food consumption explains only 0.14 (*p* > 0.1) of the diet, while urban protein-containing consumption explains 0.95 (*p* ≤ 0.01). The relationship between other food consumption and diet in urban areas was 0.51 (*p* ≤ 0.05). For the four latent variables of soybean trade, the correlation between soybean import quantity, import value, and urban-rural income and soybean trade were all above 0.95 (Table S3) and significant (*p* < 0.1). In contrast, China’s soybean production is the only latent variable with a *p* value greater than 0.1, and its correlation with trade is only 0.27. There are two possible explanations for this anomalous indicator. One of them is that China’s soybean consumption is mainly dependent on imports, as studies show that 87% of soybean consumption is dependent on imports; the second reason is that the Chinese government’s strong agricultural regulation policy may explain this phenomenon. Domestic soybean production in China is regulated more by policy than driven by dietary changes. There is also a positive covariance between these four variables, where the relationship between the negative values of the income gap and soybean production is not significant.

### Data limitations and model uncertainties

Our analysis is constrained by the fact that currently available datasets do not fully meet the specific requirements of our research, reducing their applicability to our study objectives. Firstly, the high-precision mobile phone signaling data captures short-term population movements, such as during disease outbreaks, natural disasters, or the Lunar New Year migration.^26^^,^^27^^,^^28^ However, these datasets usually have large data volumes, high computational demands, and restricted time frames, making them unsuitable for long-term trend analysis. Secondly, previous research tracked labor migration through Hukou records over time, which represents the household registration sizes in mainland China, providing insights into broad migration patterns at specific geographic scales.^29^ However, the Hukou number changes missed temporary rural-urban migration, where individuals retain their Hukou status but seek employment during crop-growing seasons. Since they are still part-time farmers, trade-influenced food prices directly affect their incomes, making them the most vulnerable to become trade-driven migrant laborers.^30^ Our model incorporates all types of mobile populations, but certain migration groups may introduce uncertainties. For example, the marriage migration, “urban-urban” migration, and “rural-urban” college students will reduce the coefficients of the “migration” variable to other related variables and will introduce uncertainties to the model. However, this impact is limited, taking “rural-urban” college students as an example, according to the “report on the survival and development of China’s mobile population”, among the mobile population, the percentage of college students (including those who have graduated) is 10.4%. According to the “report on the survival and development of China’s mobile population”, the proportion of college students (including graduates) among the mobile population is 10.4%, while the proportion of those in school age (20–29 years old) is only 1.67%. Thus, our results are solid though the agri-irrelated migration was input, the large amount of rural migration will also show the potential relationships between human mobility, diets, and global soybean trade.

Another limitation of our proposed model is that it does not account for the ability of farmers to switch to alternative crops when soybean profitability declines. Our model simplifies the economic adaptation process by assuming that reduced soybean profitability triggers rural-to-urban migration. This model overstates the relationship between soybean trade and rural-to-urban migration by failing to account for farmers’ adaptive strategies. From 2004 to 2016, of the land shifted away from soybean production in China, 70% was converted to maize, 20% to rice, 3% to vegetables, and 7% to fruits.^31^ These crop changes reflect the economic and practical considerations impacting farmers’ decisions. However, crop changes are also constrained by farmers’ knowledge, practices, and systemic agricultural limitations. In many cases, rather than switching crops, young farmers choose to migrate to urban areas. To better capture these dynamics, future studies should include the factors driving both crop substitution and rural-to-urban migration. Future studies could further investigate the bidirectional relationship between dietary transitions, soybean trade, and human mobility by employing advanced methods such as Granger causality tests or longitudinal models. These approaches would allow researchers to explore reverse causality, identifying whether changes in human mobility might also influence dietary transitions or trade dynamics. Additionally, integrating time-series data could offer deeper insights into the temporal interactions and feedback mechanisms within this system, providing a more comprehensive understanding of these complex linkages.

### Policy implications

This study uncovers an underlying mechanism where China’s dietary transition, driven by increasing consumption of protein-rich foods, has increased soybean imports. This increase in imports has lowered the expected returns for domestic soybean farmers and subsequently driven rural-to-urban migration. Based on this mechanism, we have proposed three policy suggestions for the government and policy-makers. Firstly, the rise in soybean imports has reduced the profitability of domestic soybean farming, pressuring domestic farmers to transition to non-agricultural industries, which could threaten food security by reducing domestic production. To address this, the government could provide subsidies to domestic soybean farmers and promote high-value soybean varieties to boost their market competitiveness and keep a stable agricultural workforce. Secondly, the rising demand for high-protein diets in urban populations has intensified the imbalance in soybean trade, further driving rural-to-urban migration. To address this, the government could promote healthy and sustainable dietary structures in urban populations by encouraging diverse protein sources, such as legumes, nuts, seeds, and plant-based proteins, which could reduce overreliance on imported soybeans and alleviate rural-to-urban migration pressures. Finally, the limited integration of rural areas into trade networks, coupled with the increasing demand for high-protein diets, has hindered rural economic growth and intensified labor migration to cities. To address this, the government could enhance rural infrastructure and logistics to better connect these areas to soybean trade chains, for example, by creating hubs for processing and distribution, which would boost local economic growth and help retain labor.

### Limitations of the study

There are several limitations of the existing research that must be discussed: (1) the lack of publicly available and accessible data on migrant flows may lead to some uncertainty in the data used, including the type of migration driven by multiple factors. (2) Another limitation of the model we propose is that it does not take into account the ability of farmers to switch to other crops when soybean profits decline. (3) For more discussion, please refer to the section “data limitations and model uncertainties” in the article.

## Resource availability

### Lead contact

Further information and requests for resources should be directed to and will be fulfilled by the lead contact, Dr. Ruishan Chen (rschen@sjtu.edu.cn).

### Materials availability

New datasets generated in this study have been deposited to the project public repository: https://doi.org/10.5281/zenodo.14628568.

### Data and code availability

Analyses in this study were conducted using ArcGlS Pro (Version 3.3.0), R (Version 4.3.0) and Microsoft Excel. All the data are publicly available and has been detailed in the article, Supplementary Materials, and the key resources table. Codes used in this study can be accessed from the project public repository: https://doi.org/10.5281/zenodo.14628568.

#### Data

• Data have been deposited at https://doi.org/10.5281/zenodo.14628568 and are publicly available as of the date publication. Accession numbers are listed in the key resources table.

#### Code

• All original code has been deposited at: https://doi.org/10.5281/zenodo.14628568.

Any additional information required to reanalyze the data reported in this paper is available from the lead contact upon request.

## Acknowledgments

This work was financially supported by the major program of the 10.13039/501100012456National Social Science Foundation of China (grant No. 20ZDA085), and National Key Research and Development Program of China (2023YFB3907402) . We extend our sincere gratitude to Dr. Jack (Jianguo) Liu and Dr. Emilio F. Moran for their invaluable comments and insights that greatly enhanced the quality of this paper. Their expertise and thoughtful feedback were instrumental in refining our arguments and improving the overall coherence of our work. We are deeply appreciative of their contributions to our scholarly efforts.

## Author contributions

Conceptualization and formal analysis: N.J.; methodology: N.J., and H.Y.; data curation: H.Y., Y.L., and W.S.; investigation: Y.L., W.S., R.Z., and T.M.; validation: Y.L., and W.S.; software and visualization: R.Z., and T.M.; project administration, resources, funding acquisition, and supervision: R.C.; writing – original draft: N.J., Y.L., and W.S.; writing – review and editing: H.Y., X.L., Y.L., and R.C.

## Declaration of interests

Dr. Y.S. is an editorial board member of iScience.

## STAR★Methods

### Key resources table

**Table:** 

REAGENT or RESOURCE	SOURCE	IDENTIFIER
**Deposited data**		

Zenodo includes the data and codes for this study	Zenodo	https://doi.org/10.5281/zenodo.14628567
Soybean trade data	FAO	https://www.fao.org/faostat/en/#home
Import and export data	UN ComTrade	https://comtradeplus.un.org/TradeFlow
Diets	China Statistical Yearbook (2000–2020)	https://www.stats.gov.cn/english/Statisticaldata/
Human mobility	China Statistical Yearbook (2000–2020)	https://www.stats.gov.cn/english/Statisticaldata/

**Software and algorithms**		

ArcGIS Pro 3.3.0	ESRI	https://www.esri.com/en-us/arcgis/products/arcgis-pro/overview
R 4.3.0	CRAN	https://cran.r-project.org/bin/windows/base/
Excel	Microsoft	https://www.microsoft.com/en-us/microsoft-365/excel

### Method details

#### Study area and data

In this study, China mainland (4°N-53.5°N, 73.5°E-135°E), the origin of soybeans and also the world's largest importer of soybeans, was chosen as the study area to examine the linkages between dietary transitions, soybean trade, and human mobility.^32^ References to China in this study refer specifically to mainland China, as regions such as Taiwan, Hong Kong, and Macau have independent trade and policy regimes. Considering the implementation of the reform and opening-up policy in 1978, China has experienced rapid economic growth, leading to a significant increase in demand for high-protein and high-fat foods.^33^ Benefiting from its accession to the World Trade Organization (WTO) since 2001, China's soybean imports have proliferated from 2000 to 2020, sourcing from increasingly diverse countries (Figure 3).

This study used soybean trade data at a national level to explore the relationship between dietary transitions and human mobility. Soybean exporters, such as Brazil and the United States, were identified as the sending system, while China served as the receiving system within the telecoupling framework. Annual soybean imports were treated as a primary indicator of the diet-soybean trade-human mobility relationship, reflecting how dietary transitions drive trade and influence migration patterns. These trade flows also inform decisions related to non-farm work, labor migration, planting crop alternatives, and farmland abandonment.

Data for this study were obtained from multiple sources. Soybean trade data (e.g., production, planting area, and yield) were collected from the Food and Agriculture Organization Statistics (FAOSTAT, https://www.fao.org/faostat/en/#home) for 2000–2020.^34^ Import and export data, including soybean quantities and values, were gathered from the United Nations Commodity Trade Statistics Database (UN Comtrade) using the soybean HS code 1201.^35^ Diet structure data for urban and rural populations were sourced from the China Statistical Yearbook (2000–2020),^36^ with rural diets focusing on soybean-related foods (e.g., bean products, oils, meat, egg, and milk) and urban diets including both protein-containing and non-protein foods (e.g., grains, vegetables, fruits). This is due to the fact that rural areas are mostly self-sufficient in non-protein foods and statistically insignificant.^37^ Human mobility in this study refers to the total mobile population in China, defined as individuals whose household registration (hukou) is in one location but who reside in another administrative area. This includes movements across cities, districts, or towns, as per the definition in the China Statistical Yearbook (2000–2020). The data was calculated using the 'Population by Region, Gender, and Household Registration Status' sheet.

By integrating these datasets, this study uses the telecoupling framework to link dietary transitions, soybean trade dynamics, and human mobility. The import quantities and values of soybeans were used as indicators of global trade dynamics, providing insight into how dietary transitions influence trade flows and subsequently drive migration.

#### Conceptual framework

To uncover the various nature-human interactions and relevant effects across spatial scales, the metacoupling framework was used in this study. The principle and framework of metacoupled are developed based on telecoupling's research on the interaction between distant systems which aims to qualify the impacts of distant connections such as global trade, gas emission, cropland soil erosion with distant drivers.^3^^,^^13^^,^^14^^,^^38^^,^^39^ The metacoupling framework can be treated as an umbrella framework that embodies local, adjacent, and distant scales of human-nature interactions (Figure S1). The metacoupling framework provides a comprehensive consideration of the interactions and impacts between the focal system and the adjacent systems with support through the integration of a series of interdisciplinary concepts and theories.^14^ Each metacoupled system integrates three profound complex systems through flows at local, adjacent and distant spatial scales. For instance, human-nature interactions occurring in a focal system are called intracoupling, while pericouplings are interactions that cross adjacent systems' boundaries.

Telecouplings are the human-nature interactions between distant systems. Through visualizing a typology that clarifies processes as intracoupled system, pericoupled system, and telecoupled system (Figure S2), the metacoupling framework elaborates a construction of a detailed understanding of the complexity within the socio-ecological interactions. There are five main components of each sub-framework, system, flows, causes, agents, and effects, Figure S1 is the specific structure of the intra-, peri- and telecoupled systems.

This study treats the global soybean trade system as a metacoupled system. Dietary changes within China and labor migration are treated as intracouplings and the soybean trade relation between importers and exporters can be identified as telecouplings, in our study the importer is China while the exporters are the partners of China. This trading relationship is considered one of the main soybean telecoupled systems and a key driver of decreasing labor in rural regions to migrate to urban regions in the metacoupled soybean import trade system.

We used the metacoupled framework to establish a framework to reveal the pathways that facilitate human mobility from dietary transitions. The conceptual framework includes two major interrelated components: global soybean exporters and China (importer), and the dynamics of dietary and human mobility within China. Linkages among these components constitute pathways through which diet transition is reshaping the spatial distribution of the population. We chose dietary structure in rural and urban, which represents the dynamics of food need, as the indicator of the diet with a focus on how the income gap affects protein-containing food consumption. Four indicators, responding to soybean trade, might be intermediary variables transmitting the effects of diet transition on human mobility. These include import quantity (China's total imported soybean weight), import value (China's total imported soybean value), Chinese local soybean production, and the income gap between urban and rural regions in China.

Income gap within the country will promote human mobility. Thus, we assumed that the local diet transition affects domestic human mobility through telecoupling soybean trade (Figure 4). Firstly, we assumed that trade can intensify labor migration. Considering that the concept of ‘trade’ cannot be directly observed due to its complex and multidimensional nature, we introduced a latent variable to represent ‘trade’. This latent variable is constructed using multiple observed indicators, including import quantity, import value, and soybean price, which collectively capture the economic and social dynamics associated with trade flows while accounting for measurement error. Secondly, we assumed that both the protein-containing food and non-protein-containing food consumption in both rural regions and urban regions contribute to soybean import quantity and value. However, it is essential to emphasize that protein-containing foods, such as meat, eggs, and dairy, have a much stronger and more direct relationship with soybean imports. This is because soybeans are a critical input in livestock feed production, which is necessary to meet the rising demand for protein-rich diets. Non-protein-containing foods, while included to capture broader dietary shifts, primarily reflect general agricultural market dynamics and have a more indirect influence on soybean trade. These assumptions inspired by metacoupling framework can form two-step pathways through which the diet affects the four trade indicators, which in turn affects human mobility in China.

Apart from these specified pathways, in the real world, the changing diets and increasing trade affect society through multiple pathways, such as conservative export policies that may reverse this linkage, and changing diets that affect human health and the environment.^40^^,^^41^^,^^42^ Thus, a pathway named “unspecified pathway”, which directly links diet and human mobility was also included in our framework to represent the processes that are not pointed out in this study.^43^

#### Structural equation modeling

The structural equation model (SEM) algorithm was utilized to examine the assumed linkages among diets, trade, and human mobility. Table S1 provides a detailed overview of the variables used in this study. The diet variable includes categories such as rural protein-containing foods, urban protein-containing foods, and others. Trade-related variables encompass import quantity, trade value, income gap, and the negative value of soybean production. Human mobility is represented by the total mobile population, which includes 1) rural-rural migrants, 2) rural-urban migrants, 3) urban-urban migrants, and the rural migration population. SEM has been used to quantify the predicted correlations between the variables and assess the consistency and validity of the assumed model.^44^ We do not include control variables in this article because the economic indicators considered have a high degree of collinearity with “income level” (Supplementary Material, multicollinearity analysis).

Considering the fact that only human mobility can be reasonably treated as an observed variable, with diet and trade treated as latent variables in our study, we conducted SEM modeling to verify presumed linkages among Chinese diet dynamics, global to China’s soybean trade, and human mobility, as shown in Figure 5. The specific matrix representation of the diet and trade were presented in Equations 1 and 2.

(Equation 1) $$\xi_{1}=\Lambda_{11}^{x}x_{1}+\Lambda_{21}^{x}x_{2}+\Lambda_{31}^{x}x_{3}+\delta$$

(Equation 2) $${ղ}_{1}=\Lambda_{11}^{y1}y_{1}+\Lambda_{21}^{y1}y_{2}+\Lambda_{31}^{y1}y_{3}+\varepsilon$$

where $\xi_{1}$ is the p×1 vector of exogenous variables in the model (diets in this study); ${ղ}_{1}$ represents trade, ${ղ}_{1}$ is the q×1 endogenous variables, which means the variables explained by the model; $\Lambda_{n}$ is the coefficient matrix describing the relationship with the variables; δ and ε are the error values of variables.

The general matrix expression of our SEM with two latent variables is presented in Equation 3 below:

(Equation 3) $${ղ}_{2}=B{ղ}_{1}+\Gamma \xi +\zeta$$

where ξ and ղ stand for the same variables as Equations 1 and 2; ${ղ}_{1}$ represents trade; ${ղ}_{2}$ represents human mobility; B is the q×q coefficient matrix describing the effects of endogenous variables on endogenous variables; *Γ* is the q×p coefficient matrix describing the effects of exogenous variables on the endogenous variables; ζ is the q×1 vector of errors; p is the number of exogenous variables; q is the number of endogenous variables. Because the diet and trade variables include multitudinous observed variables, we estimated the path coefficients in the model with the robust weighted least-square method.

Considering that diets in our study cannot be directly measured due to their multidimensional nature, we constructed a latent variable representing diets using multiple observed indicators, including urban protein consumption, rural protein consumption, and rural non-protein consumption. This approach allows us to capture the shared variance among these indicators, providing a more comprehensive and accurate representation of dietary transitions while accounting for measurement error:

(Equation 4) $$Y=\beta_{0}+\beta_{1}X_{1}+\beta_{2}X_{2}+\beta_{3}X_{1}X_{2}+e$$

where Equation 4 is a general regression formula for analyzing the $X_{1},X_{2}$ interaction called the mean-centered constrained approach.^45^ $\beta_{1}$ is the main effect of $X_{1}$ and $\beta_{2}$ is the main effect of $X_{2}$, $\beta_{3}$ indicates the interactions between $X_{1}$ and $X_{2}$.

The simple representation of an SEM:

(Equation 5) ψ=Bψ+Γδ+ζ

where ψ represents the endogenous variable, δ indicates the exogenous variable that is related to the endogenous ψ; B is the coefficients matrix of the expected effects of endogenous variables on exogenous variables, while Γ is the coefficients matrix of the expected effects of exogenous variables on the endogenous variables; ζ means the vector of error terms.

We used a set of validation indices to test how well the data supports the assumed pathways. All values of these indices indicated that our empirical data well supported the assumed pathways (Table S2). The annual soybean imports by China serve as key indicators of the relationship between diet, soybean trade, and human mobility. These imports are closely tied to economic factors. Four indicators, which respond to soybean trade, may act as intermediary variables in transmitting the effects of dietary transitions on human mobility. These include import quantity (total soybean weight imported), import value (total monetary value of soybeans imported), local soybean production, and the income gap between urban and rural regions in China. The data supports the model well because the selected indicators effectively represent the population segments influenced by the soybean trade. Import quantity and value capture the economic impact of dietary shifts, while local production and income gap data highlight the socioeconomic factors driving migration. This comprehensive representation ensures that the model accurately reflects the dynamics of human mobility related to the soybean trade.

After obtaining path coefficients (Table S3), we calculated the effect of each pathway through which the diet affected human mobility through trade activities (Table S4). We further calculated the net effects transmitted through trade-related indicators in this study (import quantity, trade value, income gap, and the negative value of local soybean production), the effect transmitted through unspecified processes (that is, the effect captured by the unspecified pathway), and their total (Table 1). We conducted the statistical modeling and analyses using the R package named *lavaan*.^46^

To test whether our assumed SEM model holds, we also introduced assumptions tests of the Chi-Square Goodness-of-Fit test, Dickey-Fuller test, and p-value, CFA structural validity test (CFI, RMR, and SRMR).

The rural non-protein variable was removed from the structural equation model (SEM) due to its limited statistical and theoretical contribution to the study's objectives. From a statistical perspective, the SEM results demonstrated that the rural non-protein field (Rural_Non_Prtn) did not significantly contribute to the latent variable diet. The standardized estimate for this variable was 0.201, with a high p-value of 0.281, indicating that its relationship with the diet construct was weak and non-significant. In comparison, Urban_Protein and Rural_Protein showed much stronger contributions, with standardized estimates of 0.962 (p < 0.001) and 0.582 (p = 0.001), respectively. Retaining the rural non-protein variable in the model would have introduced unnecessary noise without improving its explanatory power.

Theoretically, rural non-protein foods, such as grains and vegetables, are predominantly sourced from self-owned cultivated land in rural China. This high degree of self-sufficiency reduces their dependence on external trade, particularly the soybean trade, which is directly linked to protein-related dietary shifts. Unlike protein-rich foods, non-protein food consumption in rural areas is less influenced by external market dynamics and population mobility, making it less relevant to the research objectives. Given that this study focuses on how dietary transitions, especially those involving soybeans, drive trade and human mobility, including a variable with minimal relevance to soybean consumption would detract from the model’s interpretive clarity.

Additionally, the decision to remove this variable aligns with the goal of improving the model's overall fit and coherence. Empirical testing during the initial phases of model development consistently showed that the rural non-protein variable did not pass hypothesis tests, confirming its lack of statistical significance. The modification indices from the SEM analysis further indicated that including this variable did not enhance model fit or suggest meaningful pathways. By removing the variable, the model's chi-square statistic, degrees of freedom, and p-value were expected to improve. In the initial model, the chi-square was 31.319 with 12 degrees of freedom (p = 0.002), signaling a need for refinement.

By excluding the rural non-protein field, the refined model prioritizes variables that are more directly tied to the study's objectives. This adjustment enhances the model's statistical robustness and interpretive clarity, focusing on significant pathways between dietary transitions, trade dynamics, and human mobility. The decision reflects a rigorous and transparent process of model refinement, ensuring that the final framework aligns with the theoretical and empirical foundations of the study.

#### Multicollinearity analysis

To evaluate the suitability of including economic-related control variables in the regression model, we conducted a multicollinearity analysis using the Variance Inflation Factor (VIF). This approach helps identify potential redundancies among independent variables that could compromise the accuracy of regression coefficients and overall model reliability.

We considered several economic-related variables, including GDP, Primary Industry GDP (GDPPRI), and Agricultural GDP (Agri_Pro) (Please see https://doi.org/10.5281/zenodo.14628568 (Var_Filter.csv)), as potential control variables due to their relevance to trade dynamics, income disparities, and economic development. The analysis was conducted in R using a linear regression model with Mobility as the dependent variable.

### Quantification and statistical analysis

Statistical analyses were conducted using R (version 4.3.0) with the lavaan package for structural equation modeling (SEM). The dataset includes annual observations from 2000 to 2020 (n = 21 years). SEM was used to assess relationships among latent variables (diet and trade) and the observed variable (human mobility). Path coefficients were estimated using robust weighted least squares. Significance was assessed using p-values; p < 0.1 was considered significant. Model fit was evaluated with Chi-square, CFI, RMR, and SRMR indices (Table S2). Mean and standard deviation (SD) were used to summarize data. Statistical details, including effect sizes, n, definitions of variables, and test results, are reported in Results, Figure 2, and Tables S2–S4. All maps were created from ArcGIS Pro 3.3.0 version.
