# Supplementary material for: Global soybean trade intensifies the impacts of dietary transition on human mobility
Source: iScience. 2025 Apr 15;28(5):112426. doi: 10.1016/j.isci.2025.112426 (PMC12059661; doi:10.1016/j.isci.2025.112426)
Supplement: Document S1. Figures S1 and S2 and Tables S1–S4 [file mmc1.pdf]

iScience, Volume *28*

Supplemental information

Global soybean trade intensifies the impacts of dietary transition on human mobility

Nan Jia, Hongbo Yang, Xin Lan, Yinshuai Li, Yongze Song, Zehua Zhang, Wen Song, Rui Zhao, Tianwu Ma, and Ruishan Chen

Supplementary Information

Supplemental Figures and Legends

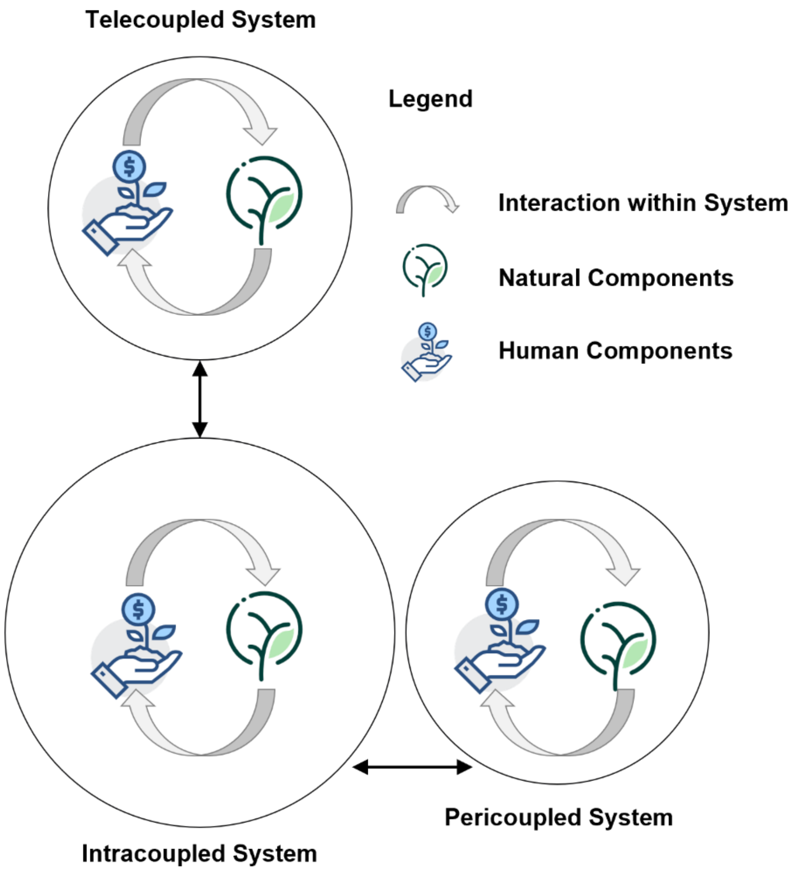

Figure S1. The three circles represent the three systems of metacoupling while the maps are the research regions of the systems. The grey arrows embody human-nature interactions, the green plants represent natural components, and the hand icon indicates human components.

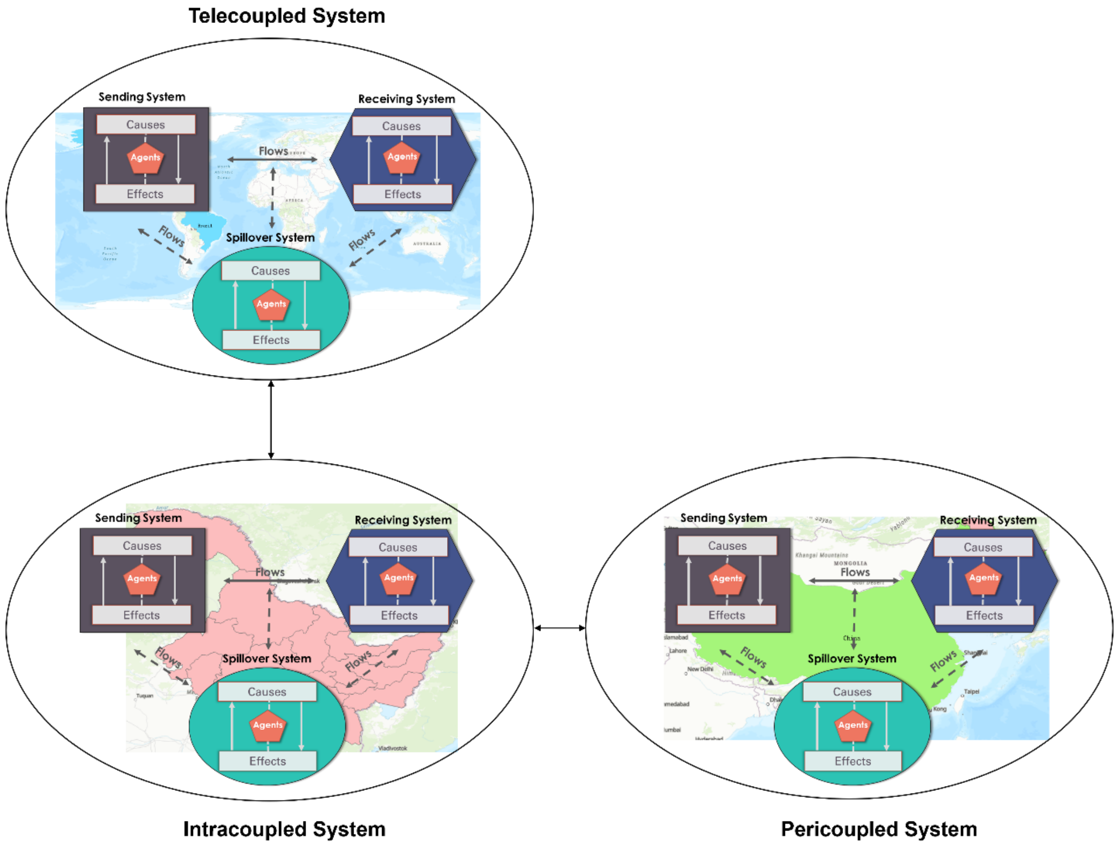

Figure S2. The conceptual metacoupling framework of this study.

Supplemental Tables

Table S1. Variables selection

Test	Paraments and valuse						
lavaan	0.6-19	ended	normally	after	102	iterations	
	Estimator	ML					
	Optimization	method	NLMINB				
	Number	of	model	parameters	16		
	Number	of	observations	22			
Model	Test	User	Model:				
	Test	statistic	31.319				
	Degrees	of	freedom	12			
	P-value	(Chi-square)	0.002				
Parameter	Estimates:						
	Standard	errors	Standard				
	Information	Expected					
	Information	saturated	(h1)	model	Structured		
Latent	Variables:						
	Estimate	Std.Err	z-value	P(>|z|)	Std.lv	Std.all	
	diet	=~					
	Urban_Protein	1	0.94	0.962			
	Rural_Protein	0.605	0.178	3.407	0.001	0.568	0.582
	Rural_Non_Prtn	0.209	0.194	1.079	0.281	0.196	0.201
	trade	=~					
	Import_Quantty	1	0.824	0.843			
	Import_Value	-1.178	0.163	-7.238	0	-0.971	-0.994
	Soybean_Price	-1.169	0.165	-7.101	0	-0.963	-0.986
	mobility	=~					
	Mobility	1	0.977	1			
Regressions:							
	Estimate	Std.Err	z-value	P(>|z|)	Std.lv	Std.all	
	trade	~					
	diet	-0.908	0.132	-6.896	0	-1.035	-1.035
	mobility	~					

	trade	-0.983	0.222	-4.429	0	-0.829	-0.829
	diet	0.141	0.144	0.983	0.326	0.136	0.136
Variances:							
	Estimate	Std.Err	z-value	P(>|z|)	Std.lv	Std.all	
	.Urban_Protein	0.072	0.042	1.718	0.086	0.072	0.075
	.Rural_Protein	0.631	0.189	3.337	0.001	0.631	0.662
	.Rural_Non_Prtn	0.916	0.274	3.345	0.001	0.916	0.96
	.Import_Quantty	0.276	0.084	3.295	0.001	0.276	0.289
	.Import_Value	0.012	0.006	2.094	0.036	0.012	0.013
	.Soybean_Price	0.027	0.009	2.907	0.004	0.027	0.028
	.Mobility	0	0	0			
	diet	0.883	0.289	3.052	0.002	1	1
	.trade	-0.048	0.035	-1.384	0.166	-0.071	-0.071
	.mobility	0.059	0.02	2.944	0.003	0.062	0.062

Table S2. Summary of validation metrics of the structural equation model

Validation statistics	Rules indicating good validation	Value for the model
Ratio of Chi-Square to df (χ^2/df)	<3	1.09
P value	>0.05	0.36
CFI (Comparative Fit Index)	>0.95	0.99
RMR (Root Mean Square Residual)	<0.08	0.06
SRMR (Standardized Root Mean Square Residual)	<0.08	0.06
RMSEA (Root Mean Square Error of Approximation)	<0.07	0.066

Table S3. Results of the latent variables. The number of years included from 2000 to 2020.

	Diet	Trade	Human mobility
Urban protein food consumption (unit: kg)	0.95 (0.09)***	/	/
Urban other food consumption (unit: kg)	0.51(0.69)**	/	/
Rural protein food consumption (unit: kg)	0.14(0.93)	/	/
Soybean import quantity (unit: million ton)	/	0.98(0.04)***	/
Soybean import valu (unit: million $)	/	0.95(0.09)**	/
Urban-Rural income gap (unit: $)	/	0.95(0.09)*	/
Chinese soybean production (unit: million $)	/	0.27(0.88)	/
Human mobility (unit: 10 million person)	/	/	1(0.0)***

*p≤ 0.1; **p≤ 0.05;***p≤ 0.001

Table S4. The effects of pathways through which the diet affected human mobility

Pathway	Effect
Diet - Trade - Human mobility	0.70**
Unspecified pathway (Diet - Human mobility)	0.09**
Total	0.79

*$p<0.05$, **$p<0.1$
